# Goodness-of-fit tests for the Compound Rayleigh distribution with application to real data

**DOI:** 10.1016/j.heliyon.2019.e02225

**Published:** 2019-08-28

**Authors:** Majdah M. Badr

**Affiliations:** Statistics Department, Faculty of Science for Girls, University of Jeddah, P. O. Box 70973, Jeddah, 21577, Saudi Arabia

**Keywords:** Applied mathematics, Computational mathematics, Statistics, Compound Rayleigh distribution, Goodness of fit test, Power function, Monte Carlo simulation, Censored sample

## Abstract

An important problem in statistics is to obtain information about the form of the population from which the sample is drawn. Goodness of fit (GOF) tests is employed to determine how well the observed sample data “fits” some proposed model. The well known standard goodness of fit tests; Kolomogorov-Smirnov (KS), Cramer von Mises (CVM) and Anderson-(AD) tests are used for continuous distributions. When the parameters are unknown, the standard tables for these tests are not valid. The complete sample procedures of goodness of fit tests are inappropriate for use with censored samples. The critical values obtained from published tables of the complete sample test statistic are necessarily conservative.

In this paper, we obtain the tables of critical values of modified Kolmogorov-Smirnov (KS) test, Cramer-Von Mises (CVM) test and Anderson-Darling (AD) test for the Compound Rayleigh (CR) distribution with unknown parameters in the case of complete and type II censored samples. Furthermore, we present power comparison between KS test, CVM test and AD test for a number of alternative distributions. Applications of the considered distribution to real medical data sets given by Stablein et al. (1981) are presented.

## 1 Introduction

Compound Rayleigh distribution is one of the models which are useful in different areas of statistics. The cumulative distribution function (cdf) and the probability density function (pdf) of (CRD) with two parameters α,βare given respectively by:

(1) $$F\left(x;\alpha ,\;\beta \right)=1-{\left(1+\frac{x^{2}}{\beta }\right)}^{-\alpha },\;\;\;\;\;\;x>0,\;\;\;\;\;\;\alpha ,\beta >0\;\;\;\;\;\;\;\;\;\;\;\;\;\;\;\;$$

(2) $${f\left(x;\alpha ,\;\beta \right)=\frac{2\alpha x}{\beta }\left(1+\frac{x^{2}}{\beta }\right)}^{-\left(\alpha +1\right)},\;\;\;\;\;\;\;\;\;x>\;0,\;\;\;\;\;\;\alpha ,\beta >0\;\;\;\;\;\;\;\;$$

Where α and β are the shape and scale parameters respectively.

Applications of CRD using a medical data are studied by Bekker et al. (2000) and Ghitany (2001). [For more details on properties of CRD(α,β), see El-Sagheer and Ahsanullah (2015) and Badr (2018)].

Goodness of fit tests are designed for a null hypothesis which is an assertion about the form of the population from which the sample is drawn. The main problem is that of testing the hypothesis about the distribution function, F(x), of the form

H_o_:F(x) = F_o_(x), where F_o_(x) = P(X≤x) is a specified family of cumulative distribution functions. When F_0_(x) is completely specified (i.e. does not contain unknown parameters) and the data are uncensored the tests are all distribution free and percentage points for the various test statistics are generally known.

The KS, CVM and AD test statistics are all distribution free and percentage points for these statistics are generally known when F_0_(x) is completely specified and the data are uncensored. This is no longer the case when data are censored or F_0_(x) involves unknown parameters. If the hypothesized distribution contains unknown parameters, then the standard tables used for these statistics no longer valid and the resulting test would be extremely conservative. In this case, these statistics can be modified by inserting estimates of parameters in F_0_(x). Hence Monte Carlo methods are often used to obtain critical values for the modified goodness of fit tests with estimated parameters.

Suppose that the distribution of X is *F*(*x*) and assume that this distribution is continuous. The EDF,*F*_*n*_(*x*), is defined by:

(3) $$F_{n}\left(x\right)=\frac{number\;of\;sample\;observations\leq x}{n},\;-\infty <x<\infty .$$

More precisely, it is defined as:

(4) $$F_{n}\left(x\right)=\{\begin{array}{c} 0,\;x<X_{\left(1\right)}\\ \frac{i}{n},\;\;\;\;\;\;\;X_{\left(i\right)}\leq x<X_{\left(i-1\right)}\\ 1,\;\;\;\;\;\;\;\;\;\;\;\;\;\;\;\;\;\;\;\;\;\;\;X_{\left(n\right)}\leq x. \end{array},\;\;i=1,\;2,\;\ldots ,\;n\;\;\;\;\;\;\;\;\;\;\;\;\;\;\;$$

Thus *F*_*n*_(*x*) is a step function, calculated from the data; as *x* increases, it takes a step up of height 1/*n* as each sample observation is reached. For any *x*, *F*_*n*_(*x*) records the proportion less than or equal to *x*, while *F*(*x*) is the probability of an observation less than or equal to *x*. We can expect *F*_*n*_(*x*) to estimate *F*(*x*), and it is in fact a consistent estimator of *F*(*x*); as n→ ∞, |*F*_*n*_(*x*)-*F*(*x*) | decreases to zero with probability one.

Several papers exist in the literatures that address the problems of finding critical values for these tests when unknown parameters which are estimated. Lilliefors (1967, 1969) had used Monte Carlo methods to construct tables for the Kolmogorov-Smirnov test when the mean and variance of a normal distribution are estimated and when the mean of an exponential distribution is estimated. Subsequent papers by Stephens (1970, 1974), Durbin (1975), and Green and Hegazy (1976) have extended the work on GOF testing for normal and exponential distribution with unknown parameters. Also, Chandra et al. (1981) studied the Kolmogorov statistics for tests of fit for the extreme-value and Weibull distributions. GOF tests for the Weibull distribution were discussed by Littell et al. (1979), Bush et al. (1983), Aho et al. (1985), Murthy et al. (2004) and Abdelfattah (2008). The paper by Woodruff et al. (1984) had addressed the problem for the gamma distribution with unknown location and scale parameters, but with known shape parameter. Moreover, Yen and Moore (1988) had discussed GOF for Laplace distribution. The exponential distribution was discussed by Balakrishnan and Basu (1995). Also, Hassan (2005) studied GOF for the generalized exponential distribution, Abd-Elfattah (2011) studied the generalized Rayleigh distribution, Wang (2008) discussed the GOF test for the exponential distribution based on progressively Type II censored sample, Allam and Zamanzade (2011) studied the case for generalized exponential distribution, Szynal and Wolynski (2012). Al-Omari and Zamanzade (2016) discussed the different goodness of fit tests for Rayleigh distribution in ranked set sampling. Zamanzade and Mohdizadeh (2017) studied the goodness of fit test for Rayleigh distribution based on Phi – divergence.

The organization of this article is as follows: Section 2 derives the maximum likelihood estimator (MLE) of the shape parameters in both complete and censored samples cases. Monte Carlo simulation is used to create tables of critical values for Kolmogorov-Smirnov (KS), Anderson-Darling (AD) and Cramer-von Mises (CvM) tests if the underlying distribution is CRD(α, β) based on type II censored sampling are provided in Section 3. In Section 4, we will study the power of these three test statistics for several values of sample size n and different alternative models based on type II censored samples. Applications to real data sets are presented in Section 5. Finally, conclusions are included in Section 6.

## 2 Methods

### 2.1 Maximum likelihood estimation

Consider a type-II censored sample$\underline{x}=\left(x_{1:n},\;x_{2:n},\;\ldots ,\;x_{r:n}\right)\;$where x_i:n_ is the time of the i^th^ component to fail. This sample of failure times is obtained and recorded from a life test of n items whose life times have a CR(α,β)distribution with density and cumulative distribution functions given, respectively, by (1) and (2). The likelihood function (LF) is:

$$\ell \left(\underline{\text{x}};\text{α},\text{β}\right)=\frac{\text{n}!}{\left(\text{n}-\text{r}\right)!}\overset{\text{r}}{\underset{\text{i}=1}{\prod }}\text{f}\left({\text{x}}_{\text{i}:\text{n}};\text{β},\;\text{α}\right){\left[1-\text{F}\left({\text{x}}_{\text{r}:\text{n}};\text{β},\;\text{α}\right)\right]}^{\text{n}-\text{r}}$$

$${{\propto {\text{α}}^{\text{r}}{\text{β}}^{-\text{r}}[\overset{\text{r}}{\underset{\text{i}=1}{\prod }}{\text{x}}_{\text{i}}(1+\frac{{\text{x}}_{\text{i}}^{2}}{\text{β}})}^{-\left(\text{α}+1\right)}].\;(1+\frac{{\text{x}}_{\text{r}}^{2}}{\text{β}})}^{-(\text{n}-\text{r})\text{α}}$$

(5) $$={\text{α}}^{\text{r}}{\text{β}}^{-\text{r}}\left(\overset{\text{r}}{\underset{\text{i}=1}{\prod }}{\text{x}}_{\text{i}})\left({\text{e}}^{-\left(\text{α}+1\right)\overset{\text{n}}{\underset{\text{i}=1}{\sum }}v_{i}}\right)({\text{e}}^{-\left(\text{n}-\text{r}\right)\text{α}v_{r}}\right),$$

where:

(6) $$v_{i}=\text{ln}\left(1+\frac{{{\text{x}}_{\text{i}}}^{2}}{\text{β}}\right),{\;\;\;\;\;\;\text{x}}_{\text{i}}={\text{x}}_{\text{i}:\text{n}},\;\;\;\;\;\;\;\;\text{i}=1,\;2,\;3,\ldots ,\text{n}\;\;\;\text{and}\;\;\;\;v_{r}=\text{ln}\left(1+\frac{{{\text{x}}_{\text{r}}}^{2}}{\text{β}}\right).\;\;$$

The logarithm of the LF is:

(7) $$\text{L}\left(\underline{\text{x}};\text{α},\text{β}\right)=\ln\;\ell \left(\underline{\text{x}};\text{α},\text{β}\right)=r\;\ln\;\alpha -r\text{ln}\beta +\overset{r}{\underset{i=1}{\sum }}\ln x_{i}-\left(\alpha +1\right)\overset{r}{\underset{i=1}{\sum }}v_{i}-\left(\text{n}-\text{r}\right)\text{α}v_{r}.\;\;$$

Differentiate (7) with respect to parametersαandβ respectively and equating the resulting to zero.

Hence, we obtain MLE's ${\hat{\alpha }}_{Mc}$ and ${\hat{\beta }}_{Mc}\;$by solving the following likelihood equations:

(8) $$\frac{r}{\alpha }-\left\{\overset{r}{\underset{i=1}{\sum }}v_{i}+\left(n-r\right)v_{r}\right\}=0\;\;\;\;\;\;\;\;\;\;\;\;\;\;\;\;\;\;\;\;$$

(9) $$\frac{-r}{\beta }+\left(\alpha +1\right)\overset{r}{\underset{i=1}{\sum }}\frac{{\text{x}}_{\text{i}}^{2}}{\beta \left(\beta +{\text{x}}_{\text{i}}^{2}\right)}+\frac{\left(n-r\right)\alpha x_{r}^{2}}{\beta \left(\beta +x_{r}^{2}\right)}=0\;\;\;\;\;\;$$

Assuming that the parameter βis known, using Eq. (8), the MLE of α, denoted by ${\hat{\alpha }}_{Mc}$, is given by

(10) $${\hat{\alpha }}_{Mc}=\frac{r}{\sum_{i=1}^{r}v_{i}+\left(n-r\right)v_{r}}.$$

In case of complete sample put r = n in (8) and (9)

The required estimates ${\hat{\alpha }}_{Mc}$ and ${\hat{\beta }}_{Mc}$ can be found by solving the two Eqs. (8) and (9) simultaneously. Clearly these equations in α and βhas no closed form analytic solutions, so, it may be solved using iterative numerical techniques such as Newton-Raphson iteration.

### 2.2 Critical values for the test statistics

In this section, we will create tables of critical values for the *KS, CVM* and AD test statistics in case of complete and censored samples if the probability distribution is Compound Rayleigh.

Suppose that we want to test the following hypothesis:

H0 The censored sample $\underline{x}=\left(x_{1:n},\;x_{2:n},\;\ldots ,\;x_{r:n}\right)\;$comes from the Compound Rayleigh distribution with unknown parameters.

H1 The distribution of the censored sample is not Compound Rayleigh distribution.

The Monte Carlo procedure for the critical values determination described in the following steps:

Step (1): Suppose that X is random variable follows Compound Rayleigh distribution, and for a fixed sample size, we generate the random sample X_1,_ X_2,_ …, X_n_ from the CR distribution with parameters α and β.

Step(2): The generated random samples are used to estimate the unknown parameters by the method of maximum likelihood as Eqs. (8) and (9).

Step(3): The resulting maximum likelihood estimators of the parameters are used to determine the hypothesized cumulative distribution function, $F_{o}\left(x;\overset{ˆ}{\alpha };\overset{ˆ}{\beta }\right)$ of the CR distribution. The appropriate test statistic was calculated for the given values of n. The test statistics were modified by replacing the unknown parameters with their maximum likelihood estimators, as follows:

1- The modified KS test statistic is
  $${\hat{D}}^{+}=\underset{1\leq i\leq r}{\max}\left\{\frac{i}{n}-{\hat{u}}_{\left(i\right)}\right\},\;$$
  $${\hat{D}}^{-}=\underset{1\leq i\leq r}{\max}\left\{{\hat{u}}_{\left(i\right)}-\frac{i-1}{n}\right\},\;\;\;\;{\hat{u}}_{\left(i\right)}=F\left(x_{i:n};\hat{Ω}\right),\;$$
  (11) $$\hat{D}=\max\left({\hat{D}}^{+}+{\hat{D}}^{-}\right)$$
2- The modified CVM test statistic is
  (12) $${{\hat{C}=\overset{r}{\underset{i=1}{\sum }}\left[{\hat{u}}_{\left(i\right)}-\frac{2i-1}{2n}\right]}^{2}+\frac{r}{12\;n^{2}}+\frac{n}{3}\left[{\hat{u}}_{\left(r\right)}-\frac{r}{n}\right]}^{3}$$
3- The modified AD test statistic is
  $$\hat{A}=\frac{-1}{n}\overset{r}{\underset{i=1}{\sum }}\left(2i-1\right)\left[ln{\hat{u}}_{\left(i\right)}-ln\left(1-{\hat{u}}_{\left(i\right)}\right)\right]-2\overset{r}{\underset{i=1}{\sum }}\ln\left[1-{\hat{u}}_{\left(i\right)}\right]$$
  (13) $$\;\;\;\;\;-\frac{1}{n}\left[{\left(r-n\right)}^{2}ln\left(1-{\hat{u}}_{\left(r\right)}\right)-r^{2}ln{\hat{u}}_{\left(r\right)}+n^{2}{\hat{u}}_{\left(r\right)}\right]$$

Thus the three test statistics were calculating for each of the samples of sizes n=5,10,15,20,30, 40 and 50. For type II censoring we used the following values of n and r.

The sizes n(r)=5(3),10(6),15(9),20(12),30(22),40(32)and50(42).Thesignificance levelusedis=0.01,0.02,0.05,0.1,0.20.

Step(4): The procedure for calculating the appropriate test statistic is repeated 5000 times. These 5000 values for KS, CMV and AD statistics were then ranked and the different quantiles were obtained. These values provided the critical values for the particular test and the sample size used, for both complete and type II censored samples.

The generated critical values for KS, CVM and AD test statistics are listed in Tables 1 and 2 when α is unknown andβ=0.5. The case when both α and β are unknown are listed in Tables 3 and 4.

**Table 1:** Critical values for KS,CVM and AD tests for complete sample when α is unknown and β = 0.5

n	Statistic	γ=0.01	γ=0.02	γ=0.05	γ=0.1	γ=0.20
*5*	*KS*	0.16236	0.17080	0.18914	0.20508	0.22838
	*CVM*	0.090846	0.09326	0.09851	0.10440	0.11353
	*AD*	0.18002	0.19199	0.22609	0.26066	0.31749
10	*KS*	0.11780	0.12409	0.13984	0.15231	0.17263
	*CVM*	0.09695	0.09997	0.10631	0.11198	0.12380
	*AD*	0.17004	0.18668	0.22562	0.26390	0.34219
15	*KS*	0.09910	0.10553	0.11526	0.12726	0.143436
	*CVM*	0.09988	0.10286	0.10839	0.11585	0.127041
	*AD*	0.16729	0.19055	0.22628	0.27518	0.35576
20	*KS*	0.08735	0.09327	0.10220	0.11314	0.12815
	*CVM*	0.10148	0.10450	0.11040	0.11884	0.13101
	*AD*	0.17193	0.19937	0.23522	0.29439	0.37895
30	*KS*	0.07351	0.07826	0.08775	0.09580	0.10934
	*CVM*	0.10330	0.10674	0.11360	0.12245	0.13660
	*AD*	0.17601	0.20486	0.25444	0.31907	0.42791
40	*KS*	0.06590	0.07017	0.07653	0.08646	0.09764
	*CVM*	0.10587	0.10995	0.11694	0.12613	0.14160
	*AD*	0.19459	0.22820	0.28338	0.35265	0.48064
50	*KS*	0.05983	0.06436	0.07174	0.08024	0.09226
	*CVM*	0.10703	0.11152	0.11885	0.13125	0.14966
	*AD*	0.20623	0.24477	0.31165	0.40422	0.57152

**Table 2:** Critical values for KS,CVM and AD tests for censored samples when α is unknown and β = 0.5

n	r	Statistic	γ=0.01	γ=0.02	γ=0.05	γ=0.1	γ=0.20
5	*3*	*KS*	0.41293	0.41644	0.42149	0.42870	0.43962
		*CVM*	0.01515	0.01547	0.01665	0.01883	0.02298
		*AD*	0.09429	0.09658	0.10486	0.12016	0.14947
10	6	*KS*	0.39310	0.395787	0.40199	0.40814	0.41731
		*CVM*	0.01022	0.0114	0.01401	0.016870	0.02202
		*AD*	0.07337	0.08131	0.10014	0.11958	0.15264
15	9	*KS*	0.38598	0.38953	0.39426	0.39986	0.40784
		*CVM*	0.00981	0.01107	0.01381	0.01707	0.02180
		*AD*	0.07316	0.08333	0.09973	0.12197	0.15761
20	12	*KS*	0.38164	0.38475	0.38986	0.39513	0.40219
		*CVM*	0.00945	0.01118	0.01374	0.01713	0.02252
		*AD*	0.07216	0.08322	0.10125	0.12629	0.16876
30	22	*KS*	0.25602	0.25958	0.26400	0.26866	0.27613
		*CVM*	0.01369	0.01615	0.02009	0.02565	0.03430
		*AD*	0.09826	0.11440	0.14322	0.18258	0.25071
40	32	*KS*	0.19670	0.19889	0.202718	0.20780	0.21506
		*CVM*	0.01733	0.02012	0.02529	0.03164	0.04301
		*AD*	0.12733	0.14530	0.18724	0.23280	0.33871
50	42	*KS*	0.16024	0.162847	0.16676	0.171574	0.17845
		*CVM*	0.01965	0.022945	0.02963	0.03832	0.05358
		*AD*	0.14626	0.165685	0.22441	0.29599	0.41962

**Table 3:** Critical values for KS,CVM and AD for complete samples when α and β are unknown.

n	Statistic	γ=0.01	γ=0.02	γ=0.05	γ=0.1	γ=0.20
*5*	*KS*	0.17719	0.18778	0.20882	0.23196	0.26571
	*CVM*	0.09655	0.09973	0.10784	0.11739	0.13477
	*AD*	0.21187	0.23378	0.28738	0.34415	0.44353
10	*KS*	0.13217	0.14140	0.15831	0.17563	0.19650
	*CVM*	0.10301	0.10886	0.11720	0.12898	0.14644
	*AD*	0.22118	0.24885	0.30707	0.38225	0.48940
15	*KS*	0.11194	0.11875	0.13178	0.14679	0.16686
	*CVM*	0.10906	0.11280	0.12242	0.13373	0.15303
	*AD*	0.24323	0.27657	0.34107	0.41648	0.53582
20	*KS*	0.10103	0.10719	0.11935	0.13142	0.14922
	*CVM*	0.11193	0.11705	0.12693	0.13917	0.16125
	*AD*	0.27647	0.30455	0.38247	0.46642	0.61164
30	*KS*	0.09028	0.09738	0.10582	0.11386	0.12950
	*CVM*	0.11611	0.12247	0.13449	0.14766	0.17236
	*AD*	0.32710	0.37696	0.46565	0.55787	0.72132
40	*KS*	0.08610	0.09297	0.10078	0.10841	0.11840
	*CVM*	0.12420	0.13078	0.14337	0.15835	0.18458
	*AD*	0.40910	0.45783	0.55675	0.66252	0.83289
50	*KS*	0.08450	0.09041	0.09899	0.10496	0.11362
	*CVM*	0.12937	0.135431	0.15108	0.16694	0.20073
	*AD*	0.49069	0.53860	0.66194	0.75937	0.97352

**Table 4:** Critical values for KS,CVM and AD for censored samples whenα and β are unknown.

n	r	Statistic	γ=0.01	γ=0.02	γ=0.05	γ=0.1	γ=0.20
5	3	*KS*	0.24859	0.26552	0.29909	0.33374	0.37654
		*CVM*	0.00647	0.00765	0.010021	0.01342	0.02063
		*AD*	0.06002	0.06671	0.08448	0.10672	0.15577
10	6	*KS*	0.21139	0.23114	0.25695	0.28609	0.32577
		*CVM*	0.0094	0.01089	0.01541	0.02052	0.03144
		*AD*	0.07331	0.08467	0.11540	0.14923	0.21758
15	9	*KS*	0.20287	0.21710	0.24611	0.27203	0.31052
		*CVM*	0.01201	0.01496	0.019134	0.02615	0.03811
		*AD*	0.09912	0.11776	0.15449	0.19959	0.27487
20	12	*KS*	0.20482	0.22069	0.24664	0.26965	0.30712
		*CVM*	0.01420	0.017527	0.02278	0.02997	0.04314
		*AD*	0.13156	0.15268	0.19211	0.24404	0.32902
30	22	*KS*	0.14374	0.15573	0.17334	0.18918	0.21360
		*CVM*	0.02602	0.02889	0.03764	0.04946	0.06893
		*AD*	0.22124	0.25276	0.31459	0.39466	0.52065
40	32	*KS*	0.11740	0.12602	0.13818	0.15177	0.16865
		*CVM*	0.03429	0.03848	0.04916	0.06487	0.090401
		*AD*	0.31366	0.35188	0.42947	0.53329	0.69027
50	42	*KS*	0.10410	0.11005	0.11888	0.12924	0.14337
		*CVM*	0.04019	0.04691	0.05957	0.07744	0.10945
		*AD*	0.40411	0.44455	0.53759	0.66065	0.84391

## 3 Analysis

### 3.1 Power comparison

The power of a goodness of fit test is defined as the probability that a statistic will lead to the rejection of the null hypothesis, H_0_, when it is false. The power of a goodness of fit test at the significance level γ is denoted by 1−β, where β is the probability of committing a type II error, failing to reject a false null hypothesis.

In this section, the power comparison was made among KS, CVM and AD statistics for the CR distribution, using some alternative distributions. These power comparisons were made using Monte Carlo simulation. We have generated 10000 pseudo-random samples of size n wheren=5,15and30 from each of the selected alternative distributions. We then calculated each of the three test statistics and compared them with their respective critical values and counted the number of rejections of the null hypothesis. The procedure was repeated for all cases of complete and censored sample sizes considered.

The alternative distributions are listed below:

1- The exponential distribution with parameter 1.5 with density
  $$\frac{1}{\theta }\exp\left(-\frac{x}{\theta }\right)$$
2- The Gamma distribution G(1.5,2) with density
  $$\frac{1}{\theta \Gamma \left(k\right)}{\left(\frac{x}{\theta }\right)}^{k-1}\;\exp\left(-\frac{x}{\theta }\right)$$
3- The Chi-square distribution with degrees of freedom $\left(x_{4}^{2}\right)$ with density
  $$\frac{1}{2^{\frac{n}{2}}\Gamma \left(\frac{n}{2}\right)}x^{\frac{n}{2}-1}\;\exp\left(-\frac{x}{2}\right)$$

Tables 5 and 6 provide the power of the *KS, CVM* and *AD* test statistics for complete or censored samples of different sample size with unknown αand β=0.5, and for the power for complete or censored samples when bothα,β are unknown.

**Table 5:** Power of KS,CVMand ADfor complete and censored samples when αis unknown and β = 0.5

Alternative	Test statistics	Sample size					
		n: 5		15		30	
		r: 5	3	15	9	30	22
*Gamma*	*KS*	0.9960	0.7424	0.9999	0.7014	1.0000	0.9799
	*CVM*	0.9979	0.9877	1.0000	0.9996	1.0000	1.0000
	*AD*	0.9967	0.9822	0.9999	0.9996	1.0000	1.0000
Chi-Square	*KS*	1.0000	1.0000	1.0000	1.0000	1.0000	1.0000
	*CVM*	1.0000	0.9710	1.0000	0.9794	1.0000	1.0000
	*AD*	1.0000	0.9261	1.0000	0.9315	1.0000	1.0000
Exponential	*KS*	0.9991	0.7600	1.0000	0.8167	1.0000	0.9777
	*CVM*	0.9991	0.8292	0.8292	0.8292	0.8292	0.8292
	*AD*	0.999	0.6422	0.6422	0.6422	0.6422	0.6422

**Table 6:** Power of KS,CVM and ADcomplete and censored samples whenα and β are unknown.

Alternative	Test statistics	Sample size					
		n: 5		15		30	
		r: 5	3	15	9	30	22
*Gamma*	*KS*	0.9921	0.9356	0.9996	0.9768	1.0000	0.9995
	*CVM*	0.9923	0.9943	0.9995	1.0000	1.0000	1.0000
	*AD*	0.9944	0.9943	0.9995	0.9994	1.0000	1.0000
Chi-Square	*KS*	1.0000	1.0000	1.0000	1.0000	1.0000	1.0000
	*CVM*	1.0000	0.9851	1.0000	0.9371	1.0000	0.9346
	*AD*	1.0000	0.9887	1.0000	0.9177	1.0000	0.9165
Exponential	*KS*	0.9961	0.9543	0.9681	0.9724	1.0000	0.9801
	*CVM*	0.9957	0.8411	0.8346	0.8411	0.8411	0.8411
	*AD*	0.9953	0.8955	0.8955	0.8955	0.8955	0.8955

The results of the power study were presented in Tables 5 and 6. It should be noted that all power comparisons were made at the 0.05 level of significance. Tables of power comparisons show that the power of the tests is quite good against all alternatives except for exponential distribution, and it gets better as the sample size increases. The powers of the tests in decreasing order are those of CvM, AD and KS tests. With no prior knowledge of the alternative distribution, it may be advisable to use the CvM statistic since its power reasonably good for all alternatives.

Next section, we apply the goodness-of-fit tests to real problems that arise in many fields of applications for the purposes of illustration study.

## 4 Methodology

### 4.1 Real data examples

In this section, we applied our proposed proceeds to real data sets for illustration purpose.

**Example 1:** Hinkley (1977) presented data consist of thirty successive March precipitation (in inches) observations. The data set is provided in the following:

**Table:** 

0.77	1.74	0.81	1.2	1.95	1.2	0.47	1.43	3.37	2.2	3	3.09	1.51	2.1
0.52	1.62	1.31	0.32	0.59	0.81	2.81	1.87	1.18	1.35	4.75	2.48	0.96	1.89
0.9	2.05												

Three distributions are fitted to this data set; Compound Rayleigh, Exponentiated Rayleigh (ER) and Rayleigh (R) distributions.

The basic descriptive statistics for the data set calculated as follows.

**Table:** 

Min	Median	Mean	Mode	Variance	Skewness	Kurtosis	Max
0.32	1.47	1.675	1.5	1.00123	1.08668	1.20688	4.75

The estimated parameters and test statistics of this data are given in Table 7.

**Table 7:** The estimated parameters and test statistics values.

Model	$\hat{\alpha }$	$\hat{\beta }$	KS	CvM	AD
CR	1.8	.63	.18	.13	.16
ER	.85	.21	.23	.21	.27
R	.82	.15	.26	.32	.22

It is observed that the CR distribution is competitive distribution compared with other distributions. In fact, based on the values of KS, CvM and AD test statistic, among the all considered fitted distributions, CR distribution is the best distribution.

**Example 2** The data set used for this example is known as average wind speed data (AWSD) and can be found in Best et al. (2010). The AWSD includes 30 average daily wind speeds (in km/h) for the month of November 2007 recorded at Elanora Heights, a north eastern suburb of Sydney, Australia. The data are as follows:

**Table:** 

2.7	3.2	2.1	4.8	7.6	4.7	4.2	4	2.9	2.9	4.6	4.8	4.3	4.6
3.7	2.4	4.9	4	7.7	10	5.2	2.6	4.2	3.6	2.5	3.3	3.1	3.7
2.8	4												

The null hypothesis is that the average wind speed follows a CRD. We can use the one – sample t – test because the number of data is 30 and approximately normal distribution as to:

From Table 8, the mean for sample is 4.17, the Sig. (2- tailed) is 0.000 this mining is high statistical significant and rejects the null hypothesis and accepts the data based on another distribution known by positive range (Gamma - Chi-Square - Exponentiated distribution) under terms mentioned. The null hypothesis is that the average wind speed don't follow CRD.

**Table 8:** One-sample test.

	Test Value = 0					
	t	df	Sig. (2-tailed)	Mean Difference	95% Confidence Interval of the Difference	
					Lower	Upper
AWSD	13.349	29	.000	4.17000	3.5311	4.8089

**Example 3**

The following original data is a subset of data reported by Bekker et al. (2000) and Stablein et al. (1981) represent the survival times in years of a group of patients given chemotherapy treatment alone. The data consisting of 46 survival times (in years) for 46 patients are: 0.047, 0.115, 0.121, 0.132, 0.164,0.197, 0.203, 0.260, 0.282, 0.296, 0.334, 0.395, 0.458, 0.466, 0.501, 0.507, 0.529, 0.534, 0.540, 0.570, 0.641, 0.644,0.696, 0.841, 0.863, 1.099, 1.219, 1.271, 1.326, 1.447, 1.485, 1.553, 1.581, 1.589, 2.178, 2.343, 2.416, 2.444, 2.825,2.830, 3.578, 3.658, 3.743, 3.978, 4.003, 4.033. We test that the data follow the Compound Rayleigh model and found it is acceptable for these data. We estimate the scale and shape parameter α and β in the CRD (α,β). Its estimated values of α and β and the corresponding test statistics are shown in the following.

The measures of the statistics indicate that the CR distribution is good fit to the real data. So, we conclude that the survival times in years of a group of patients follows CRD.

## 5 Discussion & conclusions

### 5.1 Summary and conclusion

This paper deals with constructing tables of critical values for some goodness of fit tests for Compound Rayleigh distribution based on empirical distribution functions. Several goodness of fit tests are proposed and their powers are compared with some well-known goodness of fit tests based on empirical distribution function. The Kolmogorov-Smirnov and Cramer-von Misses type goodness of fit tests as well as Anderson Darling statistics for complete and censored data were proposed. These statistics were used to goodness of fit test for CR model.

It is found that the introduced tests have good performance as compared with their competitor.

It is noted from Tables 1 and 2 that the critical values for KS,CVM and AD tests for complete were smaller than the corresponding critical values for censored samples, when is unknown and β = 0.5. It is also noted that the critical values decrease as the values of γ increase and n increase, in both cases and for the three tests.

It is also noted from Tables 3 and 4 that the critical values for KS,CVM and AD tests for complete and censored samples are smaller than the corresponding critical values for censored samples, when both α and β are both unknown. It is also noted that the critical values decrease as the values of α increase and when n increase, in both cases and for the three tests.

From Tables 5 and 6, in case of complete samples it is noted that *KS* has the smallest power values between two alternative distributions used (Gamma and Chi-Square), while *CvM* has the largest power. In case of censored samples, it is noted —in general —that *KS* has the smallest power values among the two alternative distributions used (Gamma and Chi-Square), while *CVM* has the largest power. However, when the exponential distributions used as an alternative, *KS* has the smallest power values while both *CVM* and *AD* are almost equivalent having the largest power values.

## Declarations

### Author contribution statement

Majdah M. Badr: Conceived and designed the analysis; Analyzed and interpreted the data; Contributed analysis tools or data; Wrote the paper.

### Funding statement

This project was funded by the Deanship of Scientific Research (DSR), King Abdulaziz University, Jeddah, under grant No.(G-261-363-37).

### Competing interest statement

The authors declare no conflict of interest.

### Additional information

No additional information is available for this paper.
